# The Impact of the Metal Mixture Inflammation Index on All‐Cause Mortality in Patients With Cardiovascular‐Kidney‐Metabolic Syndrome Stages 0–3: A Study Based on NHANES 1999–2010

**DOI:** 10.1155/cdr/1178167

**Published:** 2026-02-27

**Authors:** Wenlong Ding, Fachao Shi, Lei Fang, Qin Cui, Caoyang Fang

**Affiliations:** ^1^ Department of Cardiology, Xuancheng Hospital Affiliated to Wannan Medical College (Xuancheng People ′s Hospital), Xuancheng, Anhui, China; ^2^ Department of Cardiology, Maanshan People′s Hospital, Maanshan Hospital Affiliated to Wannan Medical College, Maanshan, Anhui, China; ^3^ Department of Geriatrics Center, Tongling People′s Hospital, Tongling, Anhui, China; ^4^ Department of Emergency, The First Affiliated Hospital of USTC, Division of Life Sciences and Medicine, University of Science and Technology of China, Hefei, Anhui, China, ustc.edu.cn

**Keywords:** all-cause mortality, cardiovascular-kidney-metabolic syndrome, CKM, Metal Mixture Inflammation Index, MMII, NHANES

## Abstract

**Objective:**

Environmental exposure to metals is recognized as a significant trigger of chronic inflammation and various systemic diseases, with inflammatory processes playing a central role in the development and progression of CKM syndrome. The MMII, which integrates multiple metal exposures and inflammatory responses, has demonstrated sensitivity in predicting population health risks. However, its association with all‐cause mortality in individuals with CKM Stages 0–3 has not been previously investigated.

**Methods:**

This study analyzed publicly available data from the NHANES 1999–2010, including adults aged 20 years and older with CKM syndrome Stages 0–3. The MMII was constructed based on urinary concentrations of heavy metals (mercury, cadmium, cobalt, molybdenum, lead, and tungsten) along with C‐reactive protein and white blood cell count. The primary outcome was all‐cause mortality. Cox proportional hazards regression models were employed to assess the association between MMII and all‐cause mortality, with sequential adjustment for potential confounders, complemented by sensitivity and subgroup analyses.

**Results:**

The final analysis included 2643 participants (mean age: 44.24 years; 56.77% female). Higher MMII levels were significantly associated with increased all‐cause mortality. In the fully adjusted model, participants in the highest MMII quartile exhibited a hazard ratio (HR) for all‐cause mortality of 1.50 (95% CI: 1.01–2.22, *p* = 0.04). Restricted cubic spline analysis revealed a nonlinear dose–response relationship between MMII and mortality risk (P for nonlinearity = 0.007). Threshold effect analysis identified an inflection point at MMII = 0.10594.

Threshold effect analysis identified an inflection point at MMII = 0.10594. Below this threshold, the association between MMII and all‐cause mortality was not statistically significant, with a point estimate below 1 and wide confidence intervals (HR: 0.554, 95% CI: 0.171–1.795). Above the threshold, higher MMII was associated with greater mortality risk (HR: 3.977, 95% CI: 1.777–8.901). Sensitivity analyses yielded consistent results. Subgroup analysis revealed a significant interaction between MMII and age group for mortality risk (*p* < 0.05).

**Conclusions:**

In the U.S. population with CKM Stages 0–3, MMII is significantly associated with all‐cause mortality. These findings highlight the importance of the inflammatory burden resulting from multiple metal exposures as a significant risk factor for all‐cause mortality in early‐stage CKM syndrome, underscoring the need for environmental health management and integrated approaches to chronic disease prevention.

## 1. Introduction

With the ongoing acceleration of industrialization and urbanization, environmental heavy metal pollution has emerged as an increasingly prominent global concern. Toxic metals including mercury, lead, cadmium, cobalt, molybdenum, and tungsten can enter the human body through drinking water, food, and air, subsequently accumulating in various organs and exerting deleterious effects across multiple physiological systems [1–3]. Extensive epidemiological and basic research has demonstrated that chronic, low‐dose mixed exposure to these metals can induce oxidative stress and systemic inflammatory responses, substantially contributing to the development of cardiovascular, renal, and metabolic diseases [4–6]. However, most previous studies have focused on the health effects of individual metal exposures, making it difficult to accurately assess the comprehensive health risks faced by populations exposed to multiple metals in real‐world environmental scenarios.

To address this critical gap, the Metal Mixture Inflammation Index (MMII) was developed, offering a novel tool for human risk assessment in environmental health. MMII quantitatively reflects an individual’s systemic inflammatory burden from combined metal exposures by integrating blood concentrations of various metals (including lead, mercury, cadmium, cobalt, molybdenum, and tungsten) with key inflammatory markers, such as high‐sensitivity C‐reactive protein and white blood cell counts, using weighted statistical modeling approaches [7, 8]. Compared with single‐metal analyses or individual inflammation markers, MMII demonstrates superior sensitivity in detecting the synergistic and interactive health effects of multiple metals and effectively addresses the limitations of previous single‐factor analyses. For instance, Wang et al., utilizing NHANES population data, were the first to demonstrate that elevated MMII levels are significantly associated with increased risk of diabetes, all‐cause mortality, and adverse cardiovascular outcomes, indicating that MMII provides valuable biological evidence for disease risk prediction and environmental management [7, 9]. Furthermore, as a bridge connecting complex environmental exposomics with systemic diseases, MMII has progressively emerged as a research priority in the international environmental health field.

CKM syndrome represents an innovative model of chronic disease characterized by the mutual promotion and progression of cardiovascular diseases, chronic kidney disease, and metabolic disorders such as Type 2 diabetes and obesity [10, 11]. This syndrome emphasizes that these three physiological systems share common risk factors and pathogenic mechanisms (including insulin resistance, oxidative stress, and chronic inflammation), contributing to a self‐perpetuating cycle during disease progression and significantly increasing both all‐cause and cause‐specific mortality risk [12, 13]. In recent years, major academic organizations including the American Heart Association have recognized CKM syndrome as a key focus in chronic disease management, advocating for integrated treatment approaches and multisystem risk intervention strategies in clinical practice [10]. Emerging evidence suggests that individuals with CKM syndrome are particularly susceptible to chronic low‐grade inflammation, potentially rendering them more vulnerable to the adverse effects of environmental risk factors, such as mixed metal exposure, and consequently more likely to experience poor health outcomes [14, 15].

Although the associated risks of mixed metal exposure, inflammation, and cardiovascular and metabolic diseases have garnered increasing scientific attention, systematic research examining the relationship between MMII and all‐cause mortality specifically in patients with CKM Stages 0–3 remains entirely lacking. To our knowledge, this is the first study to comprehensively evaluate the impact of MMII on all‐cause mortality in this high‐risk yet preventive‐relevant population. Furthermore, no previous research has identified a potential nonlinear threshold effect of MMII on mortality risk in any population subgroup, which could provide critical insights for precision public health interventions. Elucidating this relationship is crucial for identifying high‐risk populations, delineating the mechanistic pathway linking environmental exposure, inflammation, and chronic disease mortality, and enhancing strategies for the integrated prevention and management of environmentally influenced chronic diseases.

This study utilizes data from the NHANES 1999–2010 to systematically evaluate the impact of MMII on all‐cause mortality risk in individuals with CKM syndrome Stages 0–3. The findings provide novel epidemiological evidence illuminating the critical role of inflammatory burden under multi‐metal exposure in the prognosis of CKM‐related chronic diseases, while offering a fresh perspective for assessing health risks associated with complex environmental exposures.

While previous studies have established MMII as a predictive tool for mortality and metabolic outcomes in the general population [7, 9], its utility in multimorbid and high‐risk subgroups such as patients with CKM remains unexplored. This study extends the application of MMII beyond general population risk assessment by focusing on CKM Stages 0–3 a group representing a critical window for preventive intervention where environmental‐inflammatory interactions may be particularly deleterious. Moreover, we are the first to investigate whether a nonlinear relationship exists between MMII and mortality, which may reveal a toxicity threshold heretofore unrecognized in environmental health research.

## 2. Methods

### 2.1. Data Source and Study Object

This study employed a prospective cohort design using data from the NHANES spanning 1999 to 2010. NHANES is an ongoing cross‐sectional survey administered by the U.S. Centers for Disease Control and Prevention and the National Center for Health Statistics. It utilizes a complex, multistage probability sampling design to generate nationally representative data on the non‐institutionalized civilian population of the United States [16]. The survey gathers health‐related information through household interviews, physical examinations conducted in mobile examination centers, and laboratory tests. For this study, we utilized data encompassing demographic characteristics, health behaviors, clinical examinations, laboratory measurements, and mortality follow‐up. The original NHANES protocol was approved by the NCHS Research Ethics Review Board (Protocol #[appropriate protocol numbers for 1999‐2010 cycles: Protocol #98‐12 for the 1999‐2004 cycles and Protocol #2005‐06 for the 2005‐2010 cycles]). All participants provided informed consent. As per our institution’s policies for analyses of publicly available, de‐identified datasets, our secondary analysis was determined to be exempt from additional IRB review.

**Figure 1:**
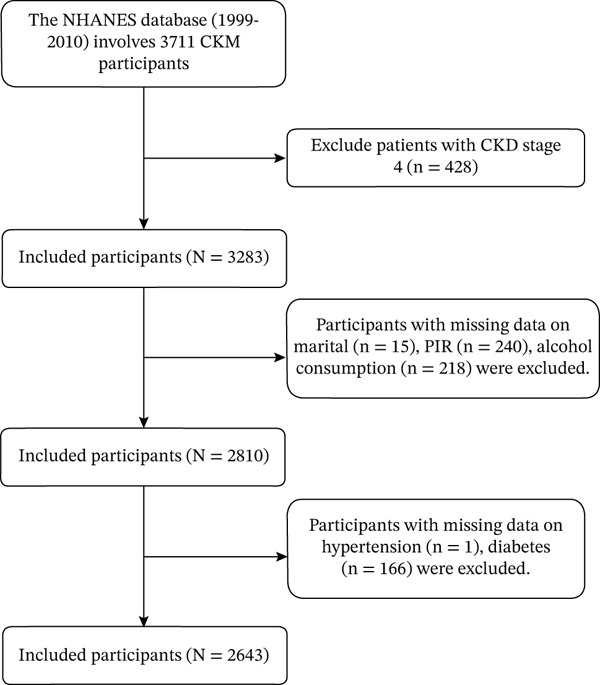
Study flow diagram.

The study population comprised adults aged 20 years or older from the NHANES 1999–2010 cycles who underwent the Mobile Examination Center (MEC) assessment and had complete data necessary for classifying CKM syndrome Stages 0–3, as well as measurements of heavy metals and inflammatory biomarkers. Pregnant women and individuals with missing key covariates or demographic information were excluded. We focused on adults with CKM Stages 0–3 because this population represents the critical window for preventive intervention where risk stratification tools are most needed. This approach allows examination of MMII’s predictive value before advanced cardiovascular disease processes dominate the clinical picture. A detailed flowchart of the study design is presented in Figure 1.

### 2.2. MMII

The MMII is a composite measure calculated according to the methodology established by Wang et al. [7], which integrates six urinary heavy metals (mercury, cadmium, cobalt, molybdenum, lead, and tungsten) with two key inflammatory biomarkers (C‐reactive protein [CRP] and white blood cell count [WBC]). To quantify the inflammatory potential of the metal mixture, we employed relative risk regression modeling and stepwise linear regression analysis [17], which enabled the identification of critical metals and assignment of specific weights based on their respective regression coefficients. The resultant MMII score provides a comprehensive assessment of systemic inflammatory burden, with elevated scores corresponding to heightened inflammatory states.

### 2.3. Definition of Cardiovascular‐Kidney‐Metabolic Syndrome (CKM)

The CKM staging was established in accordance with the latest consensus guidelines, utilizing medical history, laboratory results, and physical examination data collected in NHANES [18–20]. The staging criteria were defined as follows: CKM Stage 0: Healthy individuals without underlying diseases or risk factors. CKM Stage 1: Individuals with metabolic risk factors (including obesity, hypertension, dyslipidemia, or impaired glucose regulation) but without established diseases. CKM Stage 2: Individuals with diagnosed diabetes mellitus and/or chronic kidney disease. CKM Stage 3: Individuals with diagnosed cardiovascular disease (encompassing myocardial infarction, heart failure, stroke, or peripheral atherosclerosis).CKM Stage 4: Individuals with clinical cardiovascular disease manifestations, including coronary heart disease, heart failure, stroke, peripheral artery disease, atrial fibrillation, and other cardiovascular conditions developing on the foundation of pre‐existing CKM syndrome. Comprehensive diagnostic criteria for all stages are detailed in Table S1.

### 2.4. Outcome

The primary outcome of this study was all‐cause mortality. To ascertain vital status, participant records from NHANES 1999 to 2018 were linked to the National Death Index through probabilistic matching techniques. Because of the complex, multistage sampling design of NHANES and the variability in enrollment dates across participants, precise calculation of a median follow‐up duration was not feasible. For analytical purposes, the study endpoint was established as either the date of death or December 31, 2019, whichever occurred first.

### 2.5. Covariates

To control for potential confounding effects, we incorporated multiple covariates in our analysis, categorized as follows: Demographic characteristics: Age, sex, race/ethnicity, education level, and marital status. Lifestyle factors: Smoking status (categorized as never, former, or current smoker) and alcohol consumption (classified as never, former, heavy, moderate, or mild). Clinical indicators: Body mass index (BMI) categorized as normal (< 25 kg/m^2^), overweight (25–30 kg/m^2^), or obese (> 30 kg/m^2^).Comorbidities: Hypertension and diabetes. Medication use: Antihypertensive agents, hypoglycemic medications, and lipid‐lowering drugs. Laboratory measurements: Creatinine, uric acid, blood urea nitrogen, lymphocytes, neutrophils, monocytes, HbA1c, FPG, TG, TC, HDL, LDL, and eGFR.

### 2.6. Statistical Analysis

Considering the complex sampling design of NHANES and the inclusion of fasting biomarkers in our analysis, weighted analyses were conducted using the appropriate fasting subsample weights to account for the complex survey design and fasting subsample selection [21]. Continuous variables are presented as weighted means (SE), and categorical variables as weighted percentages (SE). MMII was categorized into four groups based on quartiles: Q1, Q2, Q3, and Q4.Because there were fewer than 5% missing values for continuous variables, we used the “MICE” package for multiple imputation to address missing values, and the proportion of missing covariates is shown in (Table S1). While some methodological concerns exist regarding multiple imputation with survey weights, we followed established approaches used in prior NHANES studies [22]. To address potential concerns, we conducted complete‐case sensitivity analyses that yielded virtually identical results (Table S4), demonstrating the robustness of our findings to different missing data handling approaches.

Weighted Cox proportional hazards regression models were employed to evaluate the association between MMII and all‐cause mortality risk. We constructed three sequential models with increasing levels of adjustment: Model 1: Unadjusted analysis; Model 2: Adjusted for demographic factors (age, sex, and race/ethnicity); Model 3: Comprehensively adjusted for age, sex, race/ethnicity, PIR, HbA1c, TG, HDL, LDL, eGFR, marital status, educational attainment, smoking status, and the use of medications for hypertension, hyperlipidemia, and diabetes HR with corresponding 95% CI were calculated for each model. To characterize potential nonlinearity, we modeled MMII as a continuous exposure using restricted cubic splines (RCS) within the survey‐weighted Cox framework. In the primary analysis, we placed five knots at the 5th, 25th, 50th, 75th, and 95th percentiles of the MMII distribution, consistent with commonly recommended practice. Nonlinearity was assessed using a Wald likelihood ratio test by comparing the spline model to a model with a single linear term for MMII. To identify a potential threshold (inflection point), we fitted a two‐piece segmented Cox model with an unknown breakpoint, estimated via an iterative profile likelihood optimization algorithm. Model fit was compared between the single‐line and the two‐line specification using a likelihood ratio test, and robust standard errors were obtained using sandwich variance estimators.

To comprehensively evaluate the robustness of our findings, we performed a series of subgroup analyses stratified by age (< 65 years vs. ≥ 65 years), BMI (< 25, 25–30, or > 30 kg/m^2^), sex, hypertension status, diabetes status, and race/ethnicity. Sensitivity analyses were conducted as follows: (1) exclusion of participants who died within the first 2 years of follow‐up; (2) exclusion of individuals with self‐reported cancer; (3) removal of participants with any missing data; and (4) multivariable logistic regression performed without incorporating survey weights. Design‐based Kaplan–Meier survival curves were estimated using survey weights (fasting subsample weight WTSAF2YR rescaled across six cycles: WTSAF12YR = WTSAF2YR/6) and design variables (SDMVSTRA, SDMVPSU). Log‐rank tests were computed using design‐adjusted procedures. All statistical analyses were performed using R (version 4.3.0), and a two‐sided *p* value < 0.05 was considered statistically significant.

## 3. Results

### 3.1. Study Population Characteristics

After applying appropriate survey weights, our analytical sample of 2643 participants represents a weighted population of approximately 114.1 million U.S. adults with CKM Stages 0–3 (mean age: 44.24 years; 56.77% female). Participants with higher MMII scores tended to be older, carried a greater clinical burden of chronic conditions including hypertension and diabetes—and were more likely to engage in adverse health behaviors such as smoking. This group also demonstrated higher all‐cause mortality during follow‐up. Detailed baseline characteristics of the study participants are provided in Table 1.

**Table 1:** Baseline characteristics of the study participants according to quartiles of MMII.

Variables	Total	Q1 (−0.735, −0.034)	Q2 (−0.034, 0.106)	Q3 (0.106, 0.267)	Q4 (0.267, 0.961)	*p* value
Age, mean (SE)	44.24 (0.48)	39.42 (0.66)	42.77 (0.66)	46.75 (0.81)	49.06 (0.83)	< 0.0001
Creatinine, mean (SE)	76.14 (0.58)	76.58 (1.15)	76.85 (1.84)	76.44 (1.09)	74.39 (0.85)	0.24
UA, mean (SE)	317.26 (1.79)	311.71 (3.47)	316.81 (3.30)	324.05 (4.49)	316.92 (3.76)	0.18
BUN, mean (SE)	4.46 (0.05)	4.50 (0.10)	4.35 (0.08)	4.58 (0.08)	4.42 (0.09)	0.18
TG, mean (SE)	1.50 (0.03)	1.50 (0.06)	1.46 (0.07)	1.53 (0.05)	1.51 (0.06)	0.82
HDL, mean (SE)	1.40 (0.01)	1.39 (0.02)	1.41 (0.02)	1.40 (0.02)	1.42 (0.02)	0.86
TC, mean (SE)	5.08 (0.02)	5.09 (0.05)	5.11 (0.05)	5.01 (0.05)	5.10 (0.06)	0.51
LDL, mean (SE)	3.02 (0.02)	3.06 (0.04)	3.06 (0.04)	2.93 (0.04)	3.02 (0.05)	0.12
PIR, mean (SE)	3.04 (0.04)	3.09 (0.07)	3.18 (0.06)	3.12 (0.08)	2.71 (0.09)	< 0.001
eGFR, mean (SE)	98.07 (0.48)	98.04 (0.94)	98.59 (0.96)	97.58 (1.01)	98.05 (1.02)	0.91
Lymphocyte, mean (SE)	2.04 (0.02)	2.07 (0.03)	2.02 (0.05)	2.06 (0.03)	2.02 (0.03)	0.46
Monocyte, mean (SE)	0.52 (0.00)	0.51 (0.01)	0.52 (0.01)	0.52 (0.01)	0.54 (0.01)	0.15
Neutrophils, mean (SE)	3.98 (0.04)	4.00 (0.08)	3.96 (0.07)	3.92 (0.07)	4.03 (0.08)	0.75
FPG, mean (SE)	5.76 (0.04)	5.78 (0.08)	5.63 (0.04)	5.79 (0.08)	5.86 (0.09)	0.02
HbA1c, mean (SE)	5.57 (0.03)	5.55 (0.04)	5.49 (0.03)	5.59 (0.05)	5.66 (0.06)	0.01
Sex, %(SE)						0.1
Female	56.77 (0.02)	51.85 (2.32)	57.93 (2.42)	58.45 (2.44)	59.45 (2.07)	
Male	43.23 (0.02)	48.15 (2.32)	42.07 (2.42)	41.55 (2.44)	40.55 (2.07)	
Race, %(SE)						< 0.0001
Mexican American	8.80 (0.01)	11.74 (1.20)	10.45 (1.40)	7.02 (1.07)	5.22 (1.02)	
Non‐Hispanic Black	11.62 (0.01)	5.68 (0.77)	10.28 (1.14)	13.39 (1.37)	18.48 (2.03)	
Non‐Hispanic White	70.89 (0.04)	71.85 (2.45)	71.90 (2.24)	72.11 (2.26)	67.13 (2.77)	
Other	8.69 (0.01)	10.72 (1.57)	7.37 (1.32)	7.47 (1.27)	9.17 (1.58)	
BMI, %(SE)						0.7
< 25	31.63 (0.01)	32.87 (1.94)	30.28 (1.91)	31.24 (2.07)	32.21 (2.29)	
25–30	35.04 (0.02)	36.62 (2.16)	35.86 (2.40)	32.85 (2.28)	34.57 (2.15)	
> 30	33.33 (0.02)	30.51 (2.28)	33.86 (2.17)	35.90 (2.55)	33.21 (2.20)	
Marital, %(SE)						< 0.0001
Divorced	10.46 (0.01)	9.45 (1.50)	6.84 (1.26)	10.39 (1.42)	16.16 (2.12)	
Married	57.22 (0.03)	54.51 (2.47)	60.57 (2.31)	58.74 (2.43)	54.74 (2.47)	
Never married	16.74 (0.01)	21.98 (2.10)	18.45 (1.95)	13.45 (1.74)	12.04 (1.43)	
Other	15.57 (0.01)	14.07 (1.78)	14.14 (1.37)	17.42 (1.74)	17.06 (1.45)	
Education, %(SE)						< 0.0001
High school or equivalent	25.52 (0.01)	23.76 (1.96)	21.98 (1.82)	26.32 (2.17)	31.04 (2.51)	
Less than high school	17.47 (0.01)	17.17 (1.30)	13.74 (1.24)	16.90 (1.78)	22.98 (2.16)	
Some college or above	57.02 (0.02)	59.07 (2.37)	64.28 (2.02)	56.78 (2.61)	45.99 (2.65)	
Smoke, %(SE)						< 0.0001
Former	23.84 (0.02)	21.07 (1.95)	22.25 (2.15)	27.19 (2.71)	25.37 (2.04)	
Never	53.43 (0.02)	66.27 (2.27)	61.26 (2.29)	49.44 (2.54)	32.81 (2.66)	
Now	22.73 (0.01)	12.66 (1.44)	16.49 (1.47)	23.37 (1.87)	41.82 (2.41)	
Alcohol, %(SE)						0.53
Former	14.40 (0.01)	12.59 (1.70)	12.66 (1.47)	15.84 (1.68)	17.08 (2.49)	
Heavy	20.87 (0.01)	21.83 (2.15)	18.80 (1.83)	21.54 (2.10)	21.46 (2.17)	
Mild	35.48 (0.02)	35.17 (1.98)	38.79 (2.24)	35.73 (2.28)	31.58 (2.31)	
Moderate	18.29 (0.01)	18.67 (1.99)	19.19 (2.01)	17.77 (2.01)	17.30 (2.44)	
Never	10.96 (0.01)	11.75 (1.73)	10.56 (1.26)	9.12 (1.28)	12.57 (1.80)	
Hypertension, %(SE)						< 0.0001
No	69.71 (0.03)	77.45 (2.19)	71.39 (2.47)	65.44 (1.89)	63.07 (2.40)	
Yes	30.29 (0.02)	22.55 (2.19)	28.61 (2.47)	34.56 (1.89)	36.93 (2.40)	
Diabetes, %(SE)						< 0.001
Borderline	12.85 (0.01)	7.92 (1.21)	13.07 (1.95)	17.09 (1.66)	13.78 (1.34)	
No	76.81 (0.03)	83.93 (1.70)	78.24 (2.16)	70.81 (1.92)	73.17 (1.84)	
Yes	10.35 (0.01)	8.15 (1.01)	8.69 (1.18)	12.09 (1.39)	13.06 (1.78)	
All‐cause mortality, %(SE)						< 0.0001
No	91.16 (0.03)	95.41 (0.73)	93.84 (1.05)	89.31 (1.39)	84.84 (1.73)	
Yes	8.84 (0.01)	4.59 (0.73)	6.16 (1.05)	10.69 (1.39)	15.16 (1.73)	
Antidiabetic, %(SE)						0.12
No	95.11 (0.03)	95.79 (0.65)	96.28 (0.72)	94.06 (1.05)	94.04 (1.09)	
Yes	4.89 (0.00)	4.21 (0.65)	3.72 (0.72)	5.94 (1.05)	5.96 (1.09)	
Antihypertensive, %(SE)						< 0.0001
No	80.32 (0.03)	88.24 (1.83)	82.10 (1.61)	75.67 (2.00)	73.77 (2.27)	
Yes	19.68 (0.01)	11.76 (1.83)	17.90 (1.61)	24.33 (2.00)	26.23 (2.27)	
Antihyperlipidemic, %(SE)						0.06
No	89.74 (0.03)	92.53 (1.29)	90.36 (1.66)	89.13 (1.70)	86.30 (1.70)	
Yes	10.26 (0.01)	7.47 (1.29)	9.64 (1.66)	10.87 (1.70)	13.70 (1.70)	

*Note:* Date are presented as mean (SE) or *n* (%); All values are weighted to represent the U.S. non‐institutionalized civilian adult population with CKM Stages 0–3. The weighted population size represented by this sample is approximately 114.1 million adults.

Abbreviations: BMI: body mass index, BUN: blood urea nitrogen, eGFR: estimated glomerular filtration rate, FPG: fasting plasma glucose, HbA1c: glycosylated hemoglobin, HDL: high density lipoprotein, LDL: Low density lipoprotein, MMII: Metal Mixture Inflammation Index; PIR: poverty income ratio, TC: Total cholesterol, TG: Triglyceride, UA: Uric acid.

### 3.2. Association of MMII With Risk of All‐Cause Mortality

During a mean follow‐up period of 148 months, we observed 333 all‐cause deaths. Multivariable Cox regression analysis revealed that in the fully adjusted Model 3, each one‐unit increment in MMII was associated with a 151% elevated risk of all‐cause mortality among patients with CKM Stages 0–3 (HR = 2.51, 95% CI: 1.30–4.84, *p* = 0.01). When comparing mortality risk across MMII quartiles, participants in the highest quartile (Q4) demonstrated a significantly increased risk of all‐cause mortality (HR = 1.50, 95% CI: 1.01–2.22, *p* = 0.04) relative to those in the lowest quartile (Q1) (Table 2).

**Table 2:** Association of the MMII and all‐cause mortality in population with CKM syndrome Stages 0–3.

Variables	Model 1		Model 2		Model 3	
	HR (95% CI)	*p*	HR (95% CI)	*p*	HR (95% CI)	*p*
**MMII**	10.88 (5.98, 19.81)	< 0.0001	4.26 (2.20, 8.26)	< 0.0001	2.51 (1.30, 4.84)	0.01
**MMIIQ**						
**Q1**	Ref	Ref	Ref	Ref	Ref	Ref
**Q2**	1.42 (0.93, 2.16)	0.11	1.10 (0.74, 1.64)	0.65	1.07 (0.73, 1.57)	0.72
**Q3**	2.55 (1.67, 3.91)	< 0.0001	1.49 (1.03, 2.17)	0.04	1.47 (0.99, 2.18)	0.06
**Q4**	3.74 (2.50, 5.59)	< 0.0001	1.93 (1.33, 2.80)	< 0.001	1.50 (1.01, 2.22)	0.04
**P for trend**	< 0.0001		< 0.001		0.021	

*Note*: Model 1: No adjustments made; Model 2: Adjusted for Age, Sex, Race; Model 3: Adjusted for Age, Sex, Race, PIR, HbA1c, TG, HDL, LDL, eGFR, Marital, Education, Smoke, Antidiabetic, Antihypertensive, Antihyperlipidemic.

Abbreviations: CKM: cardiovascular‐kidney‐metabolic; CI: confidence interval, HR: hazard ratio, MMII: Metal Mixture Inflammation Index; Ref: reference.

**Figure 2:**
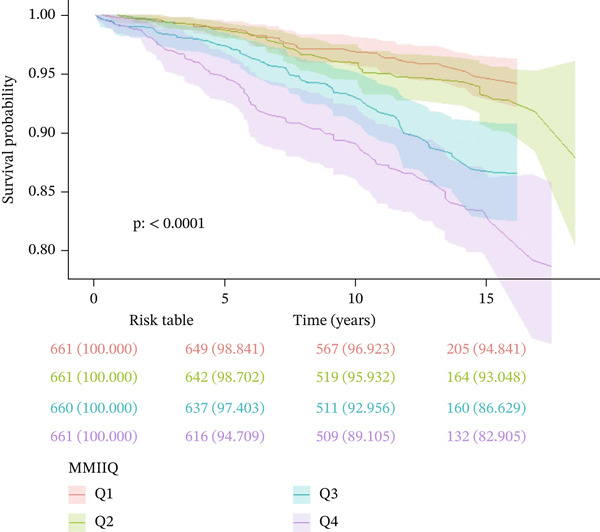
Survey‐weighted Kaplan–Meier survival curves for all‐cause mortality across MMII quartiles (Q1–Q4) in CKM Stages 0–3. Curves were estimated using the fasting subsample weight rescaled for 1999–2010 (WTSAF12YR = WTSAF2YR/6) and accounted for stratification and clustering (SDMVSTRA, SDMVPSU). The log‐rank test *p* value was obtained using a design‐adjusted procedure (*p* < 0.0001). Numbers in the risk table reflect unweighted counts for readability; survival curves and inference are survey‐weighted. CKM: cardiovascular–kidney–metabolic; MMII: Metal Mixture Inflammation Index.

Kaplan–Meier survival analysis demonstrated statistically significant differences in survival rates across the four MMII quartile groups throughout the follow‐up period (Figure 2). The lowest MMII group (Q1) consistently maintained the highest survival probability with the most gradual decline over time, whereas the highest MMII group (Q4) exhibited a steeper reduction in survival probability, indicating substantially elevated mortality risk. The between‐group survival differences were highly significant (log‐rank test, *p* < 0.0001). The risk table indicated comparable initial numbers of participants across all MMII quartile groups; however, the survival proportion diminished more rapidly in the higher MMII quartiles as follow‐up progressed. These findings demonstrate that elevated MMII levels are associated with significantly reduced survival among patients with CKM syndrome, suggesting that a higher metal mixture induced inflammatory burden may represent an independent risk marker for all‐cause mortality.

### 3.3. Dose–Response and Nonlinear Relationship

**Figure 3:**
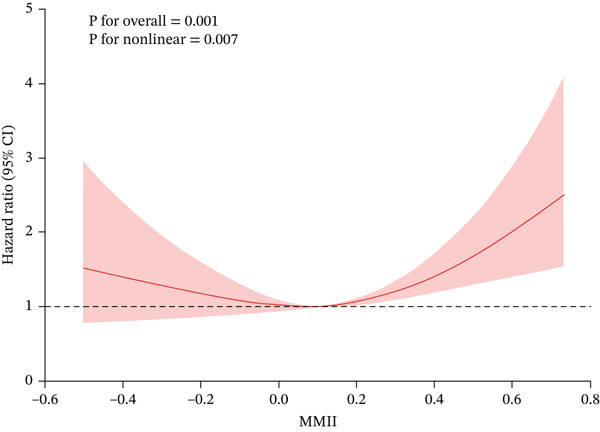
Restricted cubic spline (RCS) analysis of the association between MMII and all‐cause mortality among CKM Stages 0–3. The survey‐weighted Cox model (WTSAF12YR with SDMVSTRA and SDMVPSU) used five knots placed at the 5th, 25th, 50th, 75th, and 95th percentiles of MMII, with covariate adjustment as in Model 3. The solid line shows the estimated hazard ratio (HR) relative to the reference, and the shaded band indicates the survey‐weighted 95% confidence intervals. *p* for overall association and *p* for nonlinearity are displayed.

RCS analysis revealed a statistically significant nonlinear dose–response relationship between MMII and all‐cause mortality risk (P for nonlinearity = 0.007) (Figure 3). Subsequent piecewise regression modeling identified a distinct inflection point at MMII = 0.10594, indicating a threshold effect (Table 3). For MMII values below this threshold, we observed a negative association with mortality risk (HR = 0.554, 95% CI: 0.171–1.795); Given the nonsignificant estimate and wide confidence intervals below the threshold, we refrain from inferring a protective effect in this segment. However, this relationship was not statistically significant and was characterized by wide confidence intervals, suggesting minimal impact of MMII variations on mortality outcomes at lower exposure levels. Conversely, when MMII exceeded the threshold of 0.10594, all‐cause mortality risk increased substantially with rising MMII levels (HR = 3.977, 95% CI: 1.777–8.901, *p* < 0.01), indicating an escalation in risk associated with higher metal mixture‐induced inflammatory burden. These findings support the presence of a potential “dose‐turning point” in the relationship between MMII and all‐cause mortality among patients with CKM syndrome. This threshold effect provides valuable scientific evidence for identifying high‐risk populations and developing targeted environmental exposure intervention strategies.

**Table 3:** Threshold effect analysis for MMII and all‐cause mortality risk in CKM syndrome Stages 0–3.

	Adjusted HR(95% CI)	*p* value
Total	1.982 (1.142, 3.441)	0.0151
Segmented Cox proportional hazards model		
Inflection point	0.10594	
MMII		
< 0.10594	0.554 (0.171, 1.795)	0.3252
≥ 0.10594	3.977 (1.777, 8.901)	0.0008
*P* for Log‐likelihood ratio		0.025

HR: hazard ratio, CI: confidence interval, Ref: reference.

Model 1: No adjustments made;

Model 2: Adjusted for Age, Sex, Race;

Model 3: Adjusted for Age, Sex, Race, PIR, HbA1c, TG, HDL, LDL, eGFR, Marital, Education,

Smoke, Antidiabetic, Antihypertensive, Antihyperlipidemic. CKM: cardiovascular‐kidney‐metabolic; MMII: Metal Mixture Inflammation Index.

### 3.4. Sensitivity Analysis and Subgroup Analysis

**Figure 4:**
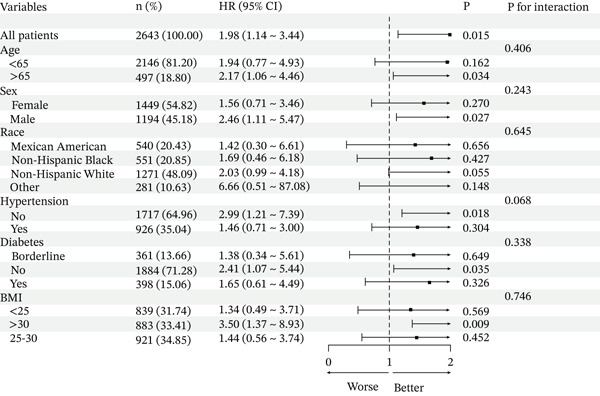
Subgroup analysis for MMII and all‐cause mortality in population with CKM syndrome Stages 0–3.Adjusted for Age, Sex, Race, PIR, HbA1c, TG, HDL, LDL, eGFR, Marital, Education, Smoke, Antidiabetic, Antihypertensive, Antihyperlipidemic. BMI: body mass index; CI: confidence interval; HR: hazard ratio.

We conducted comprehensive stratified analyses across multiple demographic and clinical subgroups, including age, sex, race/ethnicity, BMI, hypertension status, and diabetes status. No significant effect modification was observed between MMII and mortality risk across these subgroups (P for interaction > 0.05 for all stratified analyses) (Figure 4).

To assess the robustness of our findings, we performed several sensitivity analyses: (1) excluding participants who died within the first 2 years of follow‐up to mitigate potential reverse causation (Table S2, (2) excluding individuals with self‐reported cancer to eliminate the confounding effect of pre‐existing malignancy (Table S3), (3) excluding participants with any missing data to evaluate potential selection bias (Table S4); and (4) performing survey‐weighted analyses to account for the complex sampling design of NHANES (Table S5). Across all checks, results were directionally consistent with the primary survey‐weighted findings. As expected for complex survey data, unweighted models yielded slightly larger point estimates in some specifications; however, the weighted models that provide nationally representative inference under the NHANES design remained statistically significant and are the basis for interpretation.

## 4. Discussion

Based on nationally representative data from the US NHANES (1999–2010), this study systematically evaluated the association between MMII and all‐cause mortality among patients with CKM syndrome Stages 0–3. We included 2643 adults with a mean follow‐up period of 148 months. Our findings revealed that patients with higher MMII scores were generally older, carried a greater burden of chronic conditions (including hypertension and diabetes), exhibited higher prevalence of adverse lifestyle behaviors (such as smoking), and experienced elevated all‐cause mortality risk. Cox regression analysis demonstrated that each one‐unit increase in MMII was associated with a 151% higher risk of all‐cause mortality in patients with CKM Stages 0–3. Compared with the lowest quartile (Q1), participants in the highest quartile (Q4) demonstrated significantly elevated all‐cause mortality risk. Kaplan–Meier survival analysis further confirmed a progressive decline in survival probability with increasing MMII levels. Dose–response analysis using RCS models revealed a nonlinear relationship between MMII and mortality risk, with a critical threshold inflection point identified at 0.10594; above this threshold, mortality risk increased substantially. Additionally, comprehensive sensitivity and subgroup analyses yielded consistent results, supporting the robust independent effect of MMII on mortality outcomes.

Our findings align with previous research establishing strong associations between environmental heavy metal exposure, chronic low‐grade inflammation, and increased risk of cardiovascular and metabolic diseases [23–25]. For instance, Jomova et al. demonstrated that heavy metals compromise cardiovascular function and metabolic homeostasis by promoting oxidative stress and inflammatory responses [2]. Multiple studies have similarly documented that chronic metal exposure significantly correlates with increased cardiovascular morbidity and mortality [26–28]. Genuis et al. proposed that environmental toxicants disrupt immune homeostasis, thereby amplifying underlying susceptibility to multiple chronic conditions [29]. Wang et al. first introduced the MMII concept and demonstrated that elevated MMII was associated with increased risks of diabetes, kidney dysfunction, and both all‐cause and cardiovascular mortality in the general US adult population; however, their study did not specifically examine patients with CKM syndrome [7]. To our knowledge, this represents the first comprehensive assessment of the relationship between MMII and all‐cause mortality in a large cohort of patients with CKM, extending MMII applications to risk stratification in high‐risk, multimorbid populations and providing novel epidemiological evidence for the complex interactions between chronic disease states and environmental exposures.

Our detailed dose–response analysis revealed that the association between MMII and all‐cause mortality risk among patients with CKM was not simply linear but exhibited a pronounced nonlinear threshold effect. Using RCS and piecewise regression models, we identified a critical risk threshold at an MMII value of 0.10594: below this threshold, MMII demonstrated minimal impact on mortality risk, whereas above this value, all‐cause mortality risk increased markedly. This pattern suggests potential physiological tolerance to low‐level metal mixture exposure, but once the inflammatory burden exceeds this “safety threshold”, disruption of tissue and organ homeostasis precipitates substantially elevated mortality risk. These findings are consistent with research by Chowdhury et al., who similarly demonstrated nonlinear relationships between heavy metal exposure and cardiovascular disease outcomes and emphasized the importance of targeted environmental interventions focused on highly exposed populations to enable precision risk management [30]. Our results have significant clinical implications for developing targeted interventions in high‐risk chronic disease populations and establish a scientifically derived threshold that can enhance metal exposure assessment protocols and inform evidence‐based public health policy development.

Methodological rigor and clinical relevance of the threshold: Beyond documenting nonlinearity, we implemented a transparent and robust threshold identification strategy. The spline modeling followed established practices for knot placement and formally tested nonlinearity, while the segmented Cox approach estimated the breakpoint via profile likelihood and confirmed its added value over a single‐line model using likelihood ratio testing. Stability was examined using both prespecified knot‐sensitivity analyses and nonparametric bootstrap resampling, which supported the robustness of the identified threshold (MMII≈0.106). Notably, this threshold closely aligns with the Q2–Q3 boundary of MMII in our sample, indicating that mortality risk rises more steeply when inflammatory burden from metal mixtures exceeds approximately the population median. This pattern is biologically plausible given evidence that low‐level exposures may be buffered by homeostatic mechanisms, whereas surpassing a critical inflammatory load can amplify oxidative stress, inflammasome activation, endothelial dysfunction, and insulin resistance, particularly in individuals with CKM who exhibit heightened baseline inflammatory susceptibility [2, 26, 28, 31]. From a clinical and public health perspective, this threshold provides a pragmatic anchor for risk stratification and targeted exposure mitigation in CKM Stages 0–3.

Our study further underscores the distinct adverse impact of elevated MMII within high‐risk populations, particularly those with CKM syndrome. As a recently defined health model, CKM syndrome emphasizes the interconnected pathophysiology and shared risk factors among cardiovascular, renal, and metabolic systems [31–33]. Prior research has established that patients with CKM syndrome exhibit heightened baseline inflammation, significant microenvironmental dysregulation, and increased vulnerability to harmful environmental exposures [34]. Our findings not only affirm the independent prognostic value of MMII in this population but also reveal through age‐stratified analyses a pronounced risk interaction among older adults. This suggests that the detrimental “amplification effect” of MMII intensifies with advancing age and progression of CKM syndrome. Therefore, elderly individuals and those at high risk for multimorbidity should be prioritized in strategies aimed at monitoring and mitigating metal mixture exposure.

In interpreting the modest differences between weighted and unweighted estimates, it is important to note that NHANES employs stratification, clustering, unequal selection probabilities, and post‐stratification adjustments. When exposures and outcomes vary across these design features, weighting appropriately rebalances the contribution of oversampled groups (e.g., older adults, certain racial/ethnic minorities) to reflect the U.S. population structure. Consequently, weighted models may yield slightly attenuated associations relative to unweighted models, without altering substantive conclusions. Our prespecified primary analyses were survey‐weighted to ensure national representativeness and valid standard errors, whereas unweighted results were provided as sensitivity analyses to demonstrate robustness [20].

However, this study has several limitations. Firstly, as a retrospective cohort analysis, despite adjusting for multiple confounding factors, the causal relationship between MMII and all‐cause mortality risk cannot be fully established due to the inherent nature of the study design; residual confounding and reverse causality cannot be entirely ruled out. Secondly, the MMII was constructed using baseline urinary metal concentrations and inflammation markers collected at the time of the survey, which does not capture dynamic changes in exposure and inflammation over time and may underestimate or misrepresent the impact of long‐term exposure. Thirdly, the types of metals and inflammatory biomarkers included in this study were limited; some commonly encountered metals (such as arsenic and nickel) and inflammation indicators were not included, potentially leading to an incomplete assessment of metal mixture burden and inflammatory response. Fourthly, although NHANES is a nationally representative sample, the inclusion criteria and sample structure may not fully represent all racial, occupational, or highly exposed special populations, so caution is needed when generalizing these results to other countries or subgroups. Fifthly, our study used multiple imputation for handling missing data, which some methodologies note may not perfectly preserve population totals. However, our sensitivity analyses using complete‐case approaches produced identical conclusions, mitigating this concern. Sixthly, while our study was necessarily limited to NHANES 1999–2010 cycles due to CRP data availability constraints, this focused approach ensured methodological consistency in variable measurement. Future studies should validate our findings in more contemporary cohorts when compatible inflammation biomarkers become available. Lastly, potential residual confounding cannot be ruled out despite comprehensive multivariable adjustment. Our models did not include certain factors potentially related to both metal exposure and CKM outcomes, such as physical activity levels, detailed dietary patterns (particularly seafood and rice consumption that may contribute to dietary metal intake), occupational exposures, and neighborhood environmental factors. Additionally, while we adjusted for income‐to‐poverty ratio, other socioeconomic indicators like occupation type, housing characteristics, and educational quality may further refine exposure–outcome relationships. Our medication adjustment was limited to major categories without granular data on specific medications with known cardiometabolic benefits (e.g., SGLT2 inhibitors, GLP‐1 receptor agonists) or those potentially influencing metal metabolism and excretion (e.g., certain ACE inhibitors and chelating agents). These unmeasured confounders could bidirectionally influence our estimates. A key limitation of our study is the absence of external validation in an independent cohort. Future research should prioritize validating the MMII in other large‐scale population databases with available inflammatory and metabolic biomarkers. Potential validation cohorts might include the UK Biobank, the Multi‐Ethnic Study of Atherosclerosis (MESA), or the Framingham Heart Study, contingent upon the availability of comparable biomarkers. Such validation would establish the generalizability of our findings across diverse populations and strengthen the potential clinical utility of MMII as a prognostic tool. Additionally, future studies should explore whether the MMII can predict specific causes of mortality and assess its performance relative to established risk prediction tools.

In summary, our study further confirms the critical role of metal mixture‐related inflammation burden in the risk of all‐cause mortality among patients with CKM syndrome Stages 0–3, especially when exposure levels exceed specific thresholds, leading to a marked increase in mortality and adverse health outcomes. In the future, large‐scale, multicenter prospective studies and mechanistic research will help clarify the clinical translational value of MMII and provide a scientific basis for the risk identification and precise prevention of susceptible populations exposed to metal mixtures.

**Nomenclature**
BMI: Body mass index
BUN: Blood urea nitrogen
CKM: Cardiovascular‐kidney‐metabolic syndrome
CRP: C‐reactive protein
eGFR: Estimated glomerular filtration rate
FPG: Fasting plasma glucose
HbA1c: Glycosylated hemoglobin
HDL: High density lipoprotein
HR: Hazard ratio
LDL: Low density lipoprotein
MMII: Metal Mixture Inflammation Index
NHANES: National Health and Nutrition Examination Survey
PIR: Poverty income ratio
RCS: Restricted cubic spline
TG: Triglyceride
TC: Total cholesterol
UA: Uric acid

## Author Contributions

Wenlong Ding made important contributions to conception of the study, method design, and writing and editing of the manuscript. Fachao Shi and Caoyang Fang were mainly responsible for data analysis. Lei Fang and Caoyang Fang was primarily responsible for data collection. Qin Cui reviewed and edited the manuscript to ensure its scientific rigor and clarity, contributing critical input in refining the final draft.

Wenlong Ding and Fachao Shi contributed equally and are regarded as co‐first authors.

## Funding

No funding was received for this manuscript.

## Ethics Statement

The study was conducted according to the Declaration of Helsinki. All information from the NHANES program is freely available to the public and therefore does not require approval from the Medical Ethics Committee.

## Conflicts of Interest

The authors declare no conflicts of interest.

## Supporting information


**Supporting Information** Additional supporting information can be found online in the Supporting Information section. Table S1. The proportion of missing covariates. Table S2. Sensitivity analysis excluding patients who died within 2 years prior to follow‐up. Table S3. Sensitivity analyses excluded participants with self‐reported cancer diagnosis at baseline. Table S4. Sensitivity analysis excluding missing values. Table S5. Sensitivity analysis unweighted data analysis.

## Data Availability

The data that support the findings of this study are available from the corresponding author upon reasonable request.
