# Supplementary material for: The Impact of the Metal Mixture Inflammation Index on All‐Cause Mortality in Patients With Cardiovascular‐Kidney‐Metabolic Syndrome Stages 0–3: A Study Based on NHANES 1999–2010
Source: Cardiovasc Ther. 2026 Feb 27;2026:1178167. doi: 10.1155/cdr/1178167 (PMC12948730; doi:10.1155/cdr/1178167)
Supplement: Supplementary file 1 — Supporting Information Additional supporting information can be found online in the Supporting Information section. Table S1. The proportion of missing covariates. Table S2. Sensitivity analysis excluding patients who died within 2 years prior to follow‐up. Table S3. Sensitivity analyses excluded participants with self‐reported cancer diagnosis at baseline. Table S4. Sensitivity analysis excluding missing values. Table S5. Sensitivity analysis unweighted data analysis. [file CDR-2026-1178167-s001.doc]

**The Impact of the Metal Mixture Inflammation Index on All-Cause Mortality in Patients with Cardiovascular-Kidney-Metabolic Syndrome Stages 0-3: A Study Based on NHANES 1999-2010**

Wenlong Ding^1,+^,Fachao Shi^2,+^,Lei Fang^4^,Qin Cui^4,*^,Caoyang Fang^3,*^

^1^Department of Cardiology, Xuancheng Hospital Affiliated to Wannan Medical College (Xuancheng People 's Hospital), Xuancheng Anhui 242000, China;

^2^Department of Cardiology,Maanshan People's Hospital,Maanshan Hospital Affiliated to Wannan Medical College,Maanshan, Anhui,243000, China;

^3^Department of Emergency,The First Affiliated Hospital of USTC,Division of Life Sciences and Medicine, University of Science and Technology of China, Hefei, Anhui,230000, China;

^4^Department of Geriatrics Center,Tongling People's Hospital, Tongling, Anhui,244000,China;

***Corresponding Author:**

Qin Cui,Department of Geriatrics Center,Tongling People's Hospital, Tongling, Anhui,244000,China;E-mail:cuiqing1210@foxmail.com

Caoyang Fang,Department of Emergency,The First Affiliated Hospital of USTC,Division of Life Sciences and Medicine, University of Science and Technology of China, Hefei, Anhui,230000, China;E-mail:fangahslyy@foxmail.com

^+^:These authors contributed equally and are regarded as co-first authors.

**Supplementary Table S**1. The proportion of missing covariates

| **Variables** | **Proportion of missing (%)** |
| --- | --- |
| **BMI** | **0.605** |
| **Lymphocyte** | **2.459** |
| **Monocyte** | **2.459** |
| **Neutrophils** | **2.459** |
| **PFG** | **2.913** |
| **HbA1c** | **2.724** |
| **Creatinine** | **4.011** |
| **Uric acid** | **4.011** |
| **BUN** | **4.011** |
| **TG** | **3.670** |
| **TC** | **3.557** |
| **HDL** | **3.557** |
| **LDL** | **6.432** |

**BMI:Body mass index,FPG:Fasting plasma glucose,HbA1c:Glycosylated hemoglobin,BUN:Blood urea nitrogen,TG:Triglyceride,TC:Total cholesterol,HDL:High density lipoprotein,LDL:Low density lipoprotein**

**Supplementary Table S2.Sensitivity analysis excluding patients who died within 2 years prior to follow-up**

| **Variables** | **Model 1** | | **Model 2** | | **Model 3** | |
| --- | --- | --- | --- | --- | --- | --- |
|  | **HR(95%CI)** | **P** | **HR(95%CI)** | **P** | **HR(95%CI)** | **P** |
| **MMII** | 9.59(5.25,17.49) | <0.0001 | 3.80(1.99,7.28) | <0.0001 | 2.35(1.22,4.54) | 0.01 |
| **MMIIQ** | | | | | | |
| **Q1** | Ref | Ref | Ref | Ref | Ref | Ref |
| **Q2** | 1.44(0.93,2.23) | 0.10 | 1.14(0.76,1.70) | 0.54 | 1.12(0.75,1.67) | 0.58 |
| **Q3** | 2.38(1.54,3.70) | <0.001 | 1.42(0.97,2.08) | 0.07 | 1.38(0.92,2.05) | 0.12 |
| **Q4** | 3.57(2.39,5.35) | <0.0001 | 1.91(1.34,2.73) | <0.001 | 1.54(1.04,2.27) | 0.03 |
| **P for trend** | <0.0001 | | <0.001 | | 0.02 | |

**HR: hazard ratio, CI: confidence interval, Ref: reference**

**Model 1: No adjustments made;**

**Model 2: Adjusted for Age,Sex,Race;**

**Model 3:Adjusted for Age,Sex,Race,PIR,HbA1c,TG,HDL,LDL,eGFR,Marital,Education,**

**Smoke,Anti.diabetic,Anti.hypertensive,Anti.hyperlipidemic.CKM:cardiovascular-kidney-metabolic;MMII:Metal Mixture Inflammation Index**

**Supplementary Table S3. Sensitivity analyses excluded participants with self-reported cancer diagnosis at baseline**

| **Variables** | **Model 1** | | **Model 2** | | **Model 3** | |
| --- | --- | --- | --- | --- | --- | --- |
|  | **HR(95%CI)** | **P** | **HR(95%CI)** | **P** | **HR(95%CI)** | **P** |
| **MMII** | 14.57(6.90,30.78) | <0.0001 | 5.60(2.38,13.18) | <0.0001 | 3.08(1.30,7.30) | 0.01 |
| **MMIIQ** | | | | | | |
| **Q1** | Ref | Ref | Ref | Ref | Ref | Ref |
| **Q2** | 1.47(0.90,2.41) | 0.12 | 1.21(0.77,1.92) | 0.41 | 1.24(0.75,2.04) | 0.40 |
| **Q3** | 2.62(1.63,4.21) | <0.0001 | 1.66(1.10,2.52) | 0.02 | 1.52(0.97,2.38) | 0.07 |
| **Q4** | 4.35(2.69,7.02) | <0.0001 | 2.27(1.47,3.49) | <0.001 | 1.73(1.08,2.78) | 0.02 |
| **P for trend** | <0.0001 | | <0.001 | | 0.021 | |

**HR: hazard ratio, CI: confidence interval, Ref: reference**

**Model 1: No adjustments made;**

**Model 2: Adjusted for Age,Sex,Race;**

**Model 3:Adjusted for Age,Sex,Race,PIR,HbA1c,TG,HDL,LDL,eGFR,Marital,Education,**

**Smoke,Anti.diabetic,Anti.hypertensive,Anti.hyperlipidemic.CKM:cardiovascular-kidney-metabolic;MMII:Metal Mixture Inflammation Index**

**Supplementary Table S4.cSensitivity analysis excluding missing values**

| **Variables** | **Model 1** | | **Model 2** | | **Model 3** | |
| --- | --- | --- | --- | --- | --- | --- |
|  | **HR(95%CI)** | **P** | **HR(95%CI)** | **P** | **HR(95%CI)** | **P** |
| **MMII** | 11.06(5.55,22.03) | <0.0001 | 3.80(1.88,7.66) | <0.001 | 2.36(1.14,4.92) | 0.02 |
| **MMIIQ** | | | | | | |
| **Q1** | Ref | Ref | Ref | Ref | Ref | Ref |
| **Q2** | 1.37(0.87,2.18) | 0.17 | 1.03(0.68,1.57) | 0.89 | 1.04(0.68,1.61) | 0.85 |
| **Q3** | 2.47(1.57,3.89) | <0.0001 | 1.44(0.97,2.15) | 0.07 | 1.52(0.95,2.45) | 0.08 |
| **Q4** | 3.78(2.49,5.73) | <0.0001 | 1.87(1.25,2.81) | 0.002 | 1.55(1.01,2.45) | 0.04 |
| **P for trend** | <0.0001 | | <0.001 | | 0.023 | |

**HR: hazard ratio, CI: confidence interval, Ref: reference**

**Model 1: No adjustments made;**

**Model 2: Adjusted for Age,Sex,Race;**

**Model 3:Adjusted for Age,Sex,Race,PIR,HbA1c,TG,HDL,LDL,eGFR,Marital,Education,**

**Smoke,Anti.diabetic,Anti.hypertensive,Anti.hyperlipidemic.CKM:cardiovascular-kidney-metabolic;MMII:Metal Mixture Inflammation Index**

**Supplementary Table S5. Sensitivity Analysis Unweighted Data Analysis**

| **Variables** | **Model 1** | | **Model 2** | | **Model 3** | |
| --- | --- | --- | --- | --- | --- | --- |
|  | **HR(95%CI)** | **P** | **HR(95%CI)** | **P** | **HR(95%CI)** | **P** |
| **MMII** | 7.41(4.63,11.84) | <0.0001 | 3.13(1.87,5.24) | <0.0001 | 1.98(1.14,3.44) | 0.02 |
| **MMIIQ** | | | | | | |
| **Q1** | Ref | Ref | Ref | Ref | Ref | Ref |
| **Q2** | 1.34(0.93,1.94) | 0.12 | 1.07(0.74,1.54) | 0.73 | 1.07(0.74,1.55) | 0.72 |
| **Q3** | 1.79(1.27,2.54) | 0.001 | 1.07(0.75,1.52) | 0.71 | 1.06(0.75,1.51) | 0.74 |
| **Q4** | 3.00(2.17,4.14) | <0.0001 | 1.60(1.15,2.23) | 0.005 | 1.31(0.93,1.85) | 0.13 |
| **P for trend** | <0.0001 | | 0.002 | | 0.112 | |

**HR: hazard ratio, CI: confidence interval, Ref: reference**

**Model 1: No adjustments made;**

**Model 2: Adjusted for Age,Sex,Race;**

**Model 3:Adjusted for Age,Sex,Race,PIR,HbA1c,TG,HDL,LDL,eGFR,Marital,Education,**

**Smoke,Anti.diabetic,Anti.hypertensive,Anti.hyperlipidemic.CKM:cardiovascular-kidney-metabolic;MMII:Metal Mixture Inflammation Index**
